# The Italian mobile surgical units in the Great War: the modernity of the past

**DOI:** 10.1007/s13304-020-00873-9

**Published:** 2020-09-02

**Authors:** Contardo Vergani, Marco Venturi

**Affiliations:** 1 Dipartimento di Fisiopatologia Medico-Chirurgica e dei Trapianti, Università degli Studi di Milano, Milan, Italy; 2 Day Surgery Unit, Fondazione IRCCS Ca’ Granda - Ospedale Maggiore Policlinico, Via Francesco Sforza 35, Pad. Zonda, 20122 Milan, Italy

**Keywords:** World War I, Mobile health units, Ambulances, Wound and injuries, Disease outbreaks, Surgery

## Abstract

Medical services in WWI had to face enormous new problems: masses of wounded, most with devastating wounds from artillery splinters, often involving body cavities, and always contaminated. Tetanus, gas gangrene, wound infections were common and often fatal. Abdominal wounds were especially a problem: upon entering the war the commanders of all medical services ordered to avoid surgery, based on dismal experiences of previous wars. Surgical community divided into non-operative and operative treatment supporters. The problem seemed mainly organizational, as the wounded were rescued after many hours and treated by non-specialist doctors, in inadequate frontline settings or evacuated back with further delay of treatment. During initial neutrality, Italian Academics closely followed the debate, with different positions. Many courses and publications on war surgery flourished. Among the interventionists, Baldo Rossi, to provide a setting adequate to major operations close to the frontline, with trained surgeons and adequate instruments, realized for the Milano Red Cross three fully equipped, mobile surgical hospitals mounted on trucks, with an operating cabin-tent, with warming, illumination and sterilizing devices, post-operative tents and a radiological unit. Chiefs of the army approved the project and implemented seven similar units, called army surgical ambulances, each run by a distinguished surgeon. Epic history and challenges of the mobile units at the frontline, brilliant results achieved on war wounds and epidemics are described. After the war they were considered among the most significant novelties of military medical services. Parallels with present scenarios in war and peace are outlined.

## The new war

The outbreak of World War I, in August 1914, found all Armies unprepared. Army medical services were still oriented to past trends: military doctors had a general medical training, and the organization, following a hasty dressing in the field, aimed mainly at rapidly evacuating the wounded.

Soon the conflict turned into a horrific war of position. Thousands of men holed up in the trenches awaiting to go “over the top” to attempt breaking through enemy lines, despite barbed wire fences and machine-gun fire. Every attack turned into carnage. The wounded in No Man’s Land were rescued at nightfall, several hours after injury. The *Sezioni di Sanità* (comparable to the British Field ambulances) were flooded with wounded to be triaged, dressed and sent back to action if lightly wounded. Those who could be moved were evacuated to the rear field hospitals, while those who could not were treated on the spot. As a rule, no surgical operation should have been performed at the *Sezioni di Sanità* [1, 2].

Bayonet wounds were relatively infrequent, and gunshot wounds were a minority; the vast majority were devastating lacerations from artillery splinters or machine gun blasts. The onset of infection, tetanus or gas gangrene was often fatal [3].

## Do not operate on abdominal wounds!

Skull, chest and particularly abdominal wounds were a special challenge. Initially, all army medical services recommended not to operate on abdominal wounds, due to the dismal outcome of laparotomies during the Anglo-Boer war [4–7]. The famous British surgeon, Sir William MacCormac, had coined an aphorism which held true: “A man wounded in the abdomen dies if he is operated on, and remains alive if he is left in peace” [8]. Standard management consisted in nil per mouth, opiates to alleviate pain and reduce intestinal movements and semi-seated position to promote pelvic collection of pus for possible suprapubic drainage [4–6]. As this was not done in civil practice, the surgical community soon divided into abstainers and interventionists, who attributed poor results to delayed treatment carried out in hostile and inadequate settings by poorly trained and poorly equipped general doctors. This debate raged for the first 2 years [7, 8].

Italian surgeons, not yet involved in war, carefully observed the solutions adopted by the Germans and the Anglo-French. The Germans, who had penetrated far in heavily destroyed French territory, opted for building wooden surgical citadels at ten to fifteen kilometres from the frontline. The French privileged a quick evacuation network back to the nearest towns, but the wounded who could not be evacuated remained a problem. To reduce the time to intervention, the French developed the *Ambulances Chirurgicales Automobiles*: true surgical theatres mounted on trucks that could be located close to the front. The idea was good, but they soon became mammoth structures surrounded by a huge number of barracks that precluded mobility and even retreat under attack [9, 10].

## Italian surgeons prepare for war

Italian academic surgeons prepared the medical community to imminent involvement. Every University organized crash courses of war medicine and surgery, which were attended by crowds of junior and senior doctors unseen in time of peace [11]. Publications flourished [12–14]. Many Academics wrote pocket manuals on war medicine. A series of booklets “Problemi sanitari di guerra” (Health War Problems) published by Ravà & C. were sold at 10 Cents each at newspaper kiosks [11, 15]. Eminent medical leaders actively intervened in medical and political debate [16]. Among them was Baldo Rossi, the chief surgeon of Ponti and Zonda pavilions at the Ospedale Maggiore in Milano and vice-president of the local Red Cross. Taking advantage of Italian neutrality, he visited the French and German medical frontline installations, observing everything “he was allowed to observe” [17].

## The mobile surgical hospitals

He returned with the intent of developing a fully equipped Ospedale Chirurgico Mobile (Mobile Surgical Hospital) where experienced surgeons with their own teams could carry out major surgical operations [18]. The unit would be self-sufficient, producing warming, energy, illumination, sterilization. As post-operative care was considered as important as the operation itself, tents allowed the patients to be followed by the same operating team. The unit was designed to act in proximity with a *Sezione di Sanità*, which would triage patients and treat common wounds, but would refer head, chest, and abdomen injuries to the specialized Mobile Surgical Hospital [18].

**Fig. 1:**
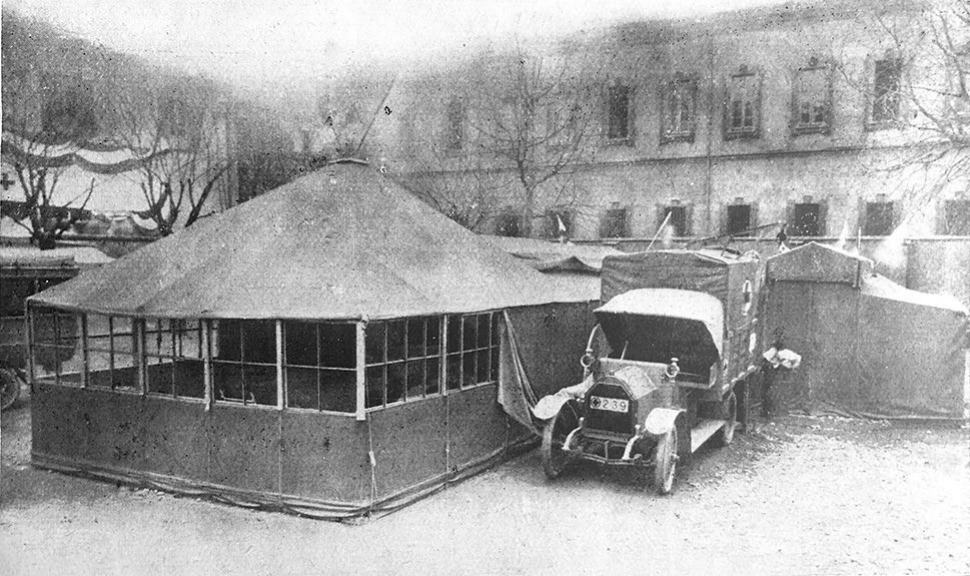
The core of the Mobile Surgical Hospital “Città di Milano”. The operating cabin-tent, connected with the two preparation tents and sided by the Fiat 15-ter truck that transported it and whose engine, once the cabin was installed, remained connected to the operating tent to provide energy for warming and illumination

Rossi and co-workers developed an operating cabin-tent that could be assembled rapidly, had wooden walls to improve wind resistance and sterility, wide windows (at that time operations were preferably performed at the natural daylight), and was covered by a double-layer tarpaulin to maintain temperature [19]. All the components were transported on a Fiat 15-ter truck. Once installed, the cabin was connected to two tents for the patient and surgeons’ preparation and for preoperative X-rays (Fig. 1). The “theatre” truck engine remained connected to the deployable theatre to provide energy for gas illumination and for sterilization Heating was achieved with portable radiators. The tents for personnel and for accommodating one hundred patients, together with the rest of the equipment were transported by six Fiat 15-ter [20]. An additional truck carried the radiologic apparatus. Once the hospital was in place, the lorries turned into ambulances to transport the wounded from the lines. The Fiat 15-ter was a slow but reliable truck that could be loaded on a standard rail car, to improve long-distance mobility [21]. The entire unit could be deployed in 6 h and repacked in four [22].

Baldo Rossi would direct the hospital with his own team from Zonda Pavilion. The hospital would be positioned right behind the lines during offensives, leaving limited personnel in Milano to cover the duties of the surgeons posted to the front-line. During pauses in the war, the proportion of personnel at home would be reversed. This was important, as mobilization of the doctors to the front line had already greatly impoverished civil medical assistance at home.

## The fund-raising campaign

Back from the western front, Rossi carried out a veritable communication campaign, holding conferences to medical and lay audiences and writing articles in specialized and common press, to sensitize citizens and administrators of Milano [23–25].

He was well introduced at the Military Headquarter, and presented his detailed project to general Porro, who was Cadorna’s second in command [20, 22]. The project was approved. Rossi set up a “Committee for Mobile Surgical Hospitals” with many influent personalities and launched a fundraising campaign on the columns of the *Secolo* and *Corriere della Sera* at the end of December 1915. Bankers, entrepreneurs, merchants and professionals, as well as common people, responded generously. In few days he collected about 230.000 lire: enough to build and equip the first mobile unit [26, 27]. It was a huge amount of money, equivalent to about 850,000 euro today [28], but with a far greater purchasing power The entire “ultramodern” Zonda Pavilion had cost 300.000 lire the same year.

**Fig. 2:**
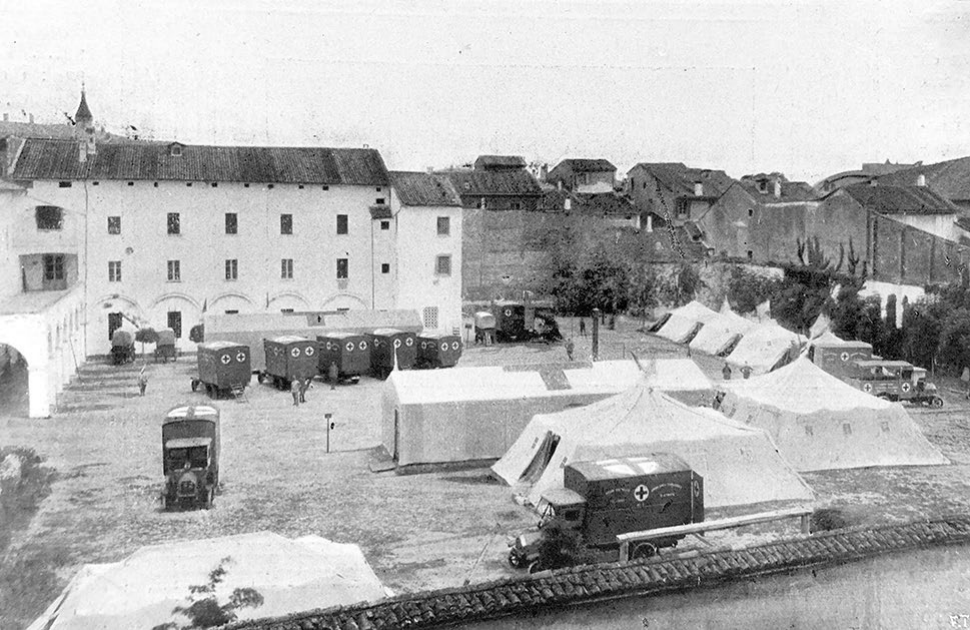
An Army Surgical Ambulance deployed in the courtyard of Caserma Masino in Bologna in May 1916 before departing for the frontline. The long rectangular tent hosted the theatre and X-rays apparatus

**Fig. 3:**
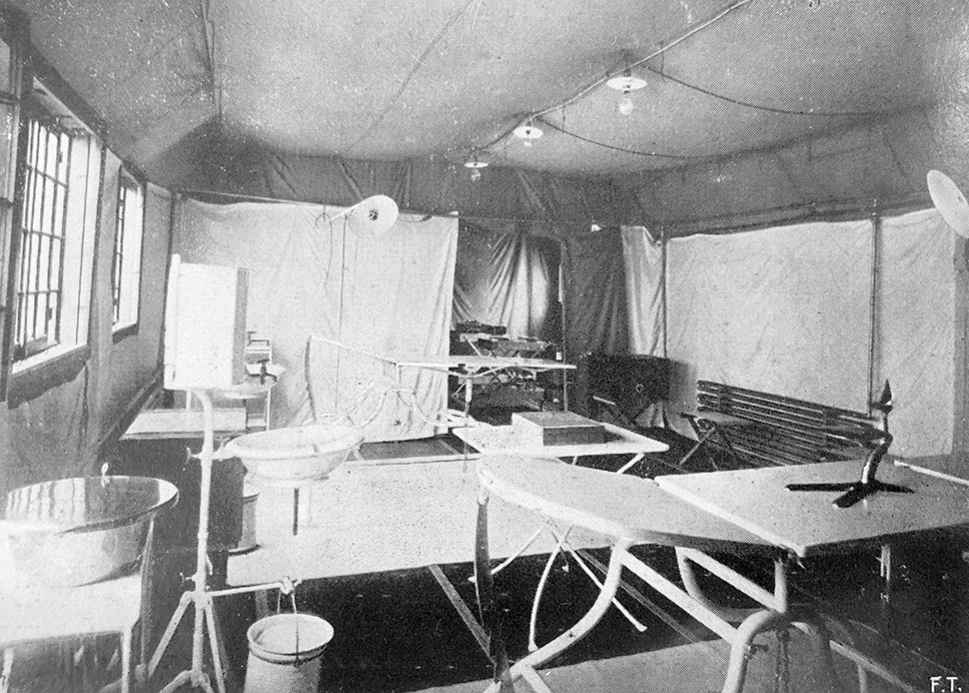
The interior of the theatre of an Army Surgical Ambulance. Notice the twin-beds system, electric lamps hanging from the roof, and a warming radiator on the right side

Every day the press reported the list of donors with the sum they donated, to trigger emulation. True to expectations, the first unit was called Ospedale Chirurgico Mobile “Città di Milano”. Rossi was aware that a single unit was not enough, and convinced the Charity Committee of the local bank to finance a second unit therefore named “Cassa di Risparmio delle Provincie Lombarde” and entrusted to professor Bozzi from Genoa [22, 29]. The Commanders of the Military Medical Corps then enthusiastically assigned a Committee of eminent professionals in Bologna the task of implementing five well-equipped *Ambulanze Chirurgiche d’Armata* (Army Surgical Ambulances*).* These were analogous to Mobile Surgical Hospitals (Fig. 2), but had a capacity of only 24–48 post-operative beds, a larger operating tent with smaller windows, and electric illumination instead of gas lamps (Fig. 3). Each Unit was transported on eight Fiat 18BL, which were heavier than Fiat 15-ter [30]. The Ambulances were entrusted to distinguished surgeons from major Italian Universities and hospitals of the time.

## The mobile surgical hospitals go to war

The Ospedale Mobile “Città di Milano” was inaugurated in March 1916, and displayed for the people of Milano, who had financed it [20, 22]. As a further innovation Baldo Rossi, who had witnessed women’s contribution to caring for the sick at the Ospedale Maggiore, strongly supported and obtained the posting of four volunteer Red Cross women nurses to every deployed mobile unit: a debated decision at the time [22].

**Fig. 4:**
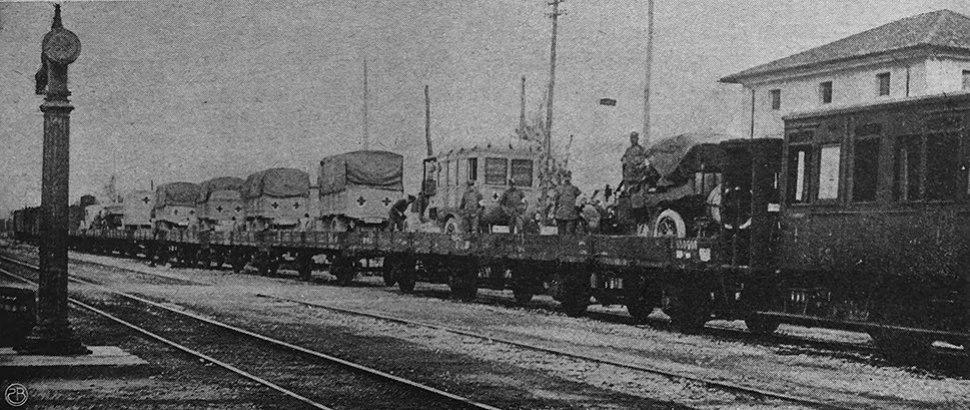
The long convoy of trucks loaded on the train that transported the two Red Cross Mobile Surgical Hospitals to the frontline

On May 16th, the two hospitals “Città di Milano” and “Cassa di Risparmio delle Provincie Lombarde” were loaded on a long rail convoy headed to the Isonzo front [31] (Fig. 4).

## On the mountain front during the *Strafexpedition*

**Fig. 5:**
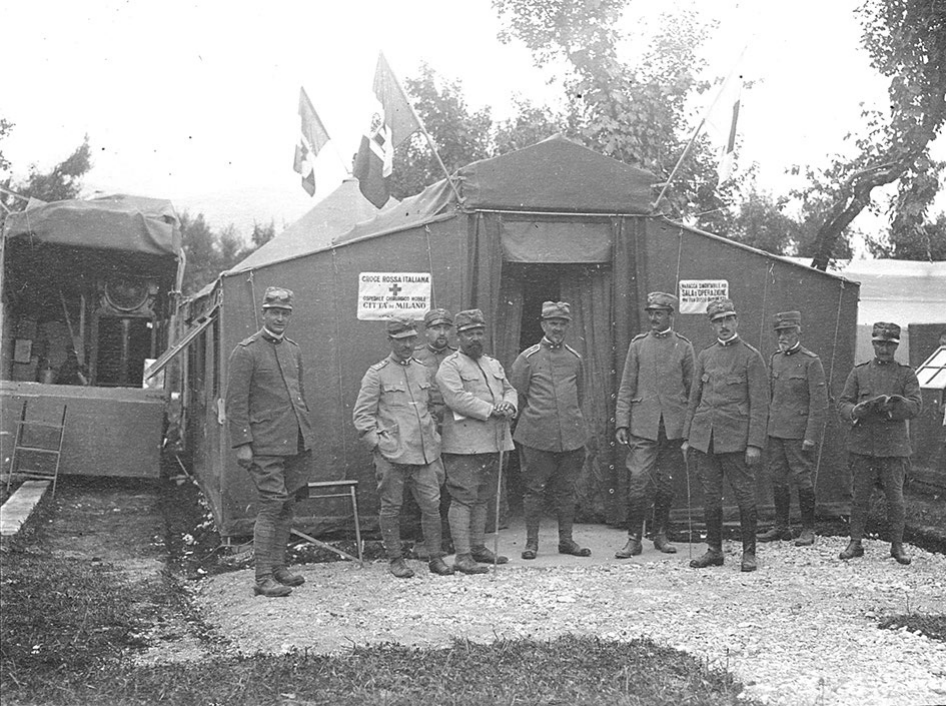
Professor Rossi (in the centre of the picture) and his collaborators in front of the “core” of the Mobile Surgical Hospital “Città di Milano” installed in Mason Vicentino. June 1916 (Courtesy Insmli—Milano Fondo Solaro A00_03264_0002_010_001r)

**Fig. 6:**
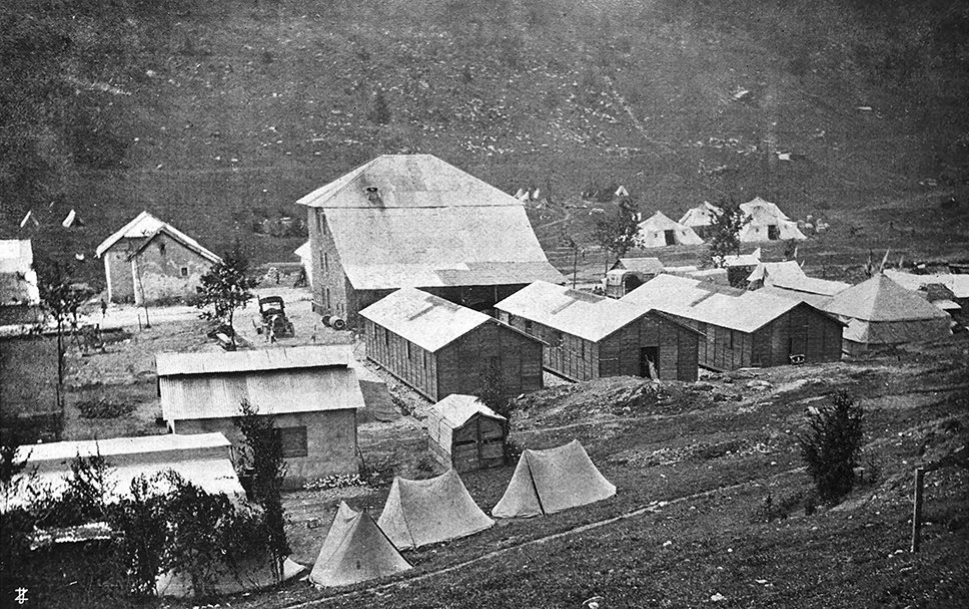
The Mobile surgical Hospital Città di Milano deployed at Campi Mezzavia in the hearth of the Asiago Plateau. The hospital was accomodated in preexisting buildings, in tents and in barracks, but operations were always performed in the movable operating cabin-tent (visible on the right side of the picture past the third wooden barrack)

Since just in those days Austria had launched the *Strafexpedition* in Veneto and Trentino, while the Isonzo front was quiet, professors Rossi and Bozzi asked to be redeployed on the “hot” front, and were sent to Ala (Trentino, at the mouth of the Adige Valley) and Schio (Veneto, close to the Mount Pasubio) respectively. The first completed Army Surgical Ambulances were temporarily posted to the Veneto rear-front at Cittadella (First Surgical Ambulance) and Thiene (Third Surgical Ambulance). The mobile units could not be installed close to the combat line due to the mountain terrain and scarcity of water, and this limited their efficacy in treating penetrating wounds. At the end of June an attempt of deploying the third Army Surgical Ambulance on the Asiago Plateau failed due to impossible logistic conditions; the same difficulties encountered the fourth Army Surgical Ambulance, posted to Falcade at 1200 m of altitude at the border between Veneto and Trentino. Later in June the front changed, allowing the “Città di Milano” to be redeployed at the foot of the Asiago plateau. In 24 h the entire hospital was dismantled, loaded on trucks and train, transported and set up in Mason Vicentino, 150 km away (Fig. 5). Mobility had been definitely demonstrated; efficacy still remained to be proven. By mid-July an advanced section was set up at Campi di Mezzavia, in the heart of the plateau and much closer to the combat lines. Surgical results greatly improved [22, 31] (Fig. 6).

Following this early experience, a harsh controversy on the role and efficacy of the expensive mobile units was reported on the three leading Italian medical journals of the time: “Policlinico”, “L’Ospedale Maggiore” and “Rivista Ospedaliera”, which also hosted the discussion between non-operative treatment supporters and interventionists, echoing the parallel French debate [32, 33].

Once Italians had conquered Gorizia early in August, six out of the seven mobile surgical units were redeployed along the Isonzo front. The five Army Surgical Ambulances and the two Red Cross Mobile Surgical Hospitals acted independently one from the other, under the orders and coordination of the Medical Corps Headquarter, but the seven directors maintained a close interconnection. In November military authorities, and the directors of the mobile units met to evaluate the first six months of activity [34]. They decided to add a small and divisible operating cabin (the “Ambulanzetta”, i.e. little ambulance), to permit advancing sections of the main unit, as allowed by the terrain, and determined to implement two additional Army Ambulances [34]. Milano Red Cross was already equipping a third Mobile Surgical Hospital. The unit had also been funded by Cassa di Risparmio, and was named after the great Milanese surgeon Giovambattista Monteggia, in the centennial of his death [22].

## The XXV Congress of the Italian Society of Surgery

In spring 1917, the Italian Society of Surgery hold its XXV Congress in Bologna. Considering the extreme interest of surgical topics even for non-surgeons, the Military had promoted participation of a large number of medical officers. The organizing committee had received more than one hundred and fifty communications, which were grouped in six thematic sessions, covering every aspect of war surgery. Each saw a significant contribution by the specialized mobile surgical units. The directors of the mobile units presented their preliminary results on more than 1100 war abdominal operations, reporting very encouraging survival rates, ranging between 20 and 36% in patients who would have almost surely died. As during the Societé Francaise Congress a few months earlier, numbers ratified the superiority of operative treatment. Despite positive results, some academic surgeons expressed heavy criticisms toward mobile units, but many others, including Edoardo Bassini, father of the revolutionary groin hernia repair and a former *garibaldino,* reacted positively. The controversy continued on medical journals [35].

## A crucial year: 1917

Aiming to open the road to Wien, Italians prepared an offensive to conquer the Bainsizza plateau early in 1917.

The “Città di Milano” was in Quisca, on the hills west of the Isonzo river, near the crossings points to the Bainsizza plateau, and, thanks to its mobile conception, was able to set an advanced section with theatre, X-Rays room and ward in a railway tunnel, in front of Zagora hill, a site of fiercely fought actions. Southwards, at Gradisca, the 4th Army Ambulance similarly organized an advanced section and theatre in the Devetachi caves, one of the Carso most dangerous sites. At Zagora and Devetachi surgeons and nurses wrote pages of courage and dedication while operating under heavy bombings [37].

The “Cassa di Risparmio”, and the other Army Surgical Ambulances were disseminated behind Gorizia and the Carso plateau, while the third mobile hospital “Monteggia” had started its activity in the Judrio valley in July, on the path that the troops marching towards the Bainsizza plateau would take [22, 31, 36].

Once conquered the Bainsizza, the Zagora section again moved to Ravne, another “hot” site”, and for a couple of months was the only specialized surgical unit in the entire plateau, deserving a warm tribute by the famous British historian Trevelyan, who was in command of the local British Red Cross Motor Ambulances and a direct witness of medics' courage in Ravne [21].

## Caporetto

The Austrian-German forces broke the front at Caporetto on October 24th. The “Monteggia” was quickly reached and destroyed, but all the patients had been safely evacuated thanks to the courage of the doctors and of the four Red Cross nurses [36]. The Ravne unit, though damaged by enemy bombs, managed to re-join the mother unit in Quisca. The “Città di Milano” evacuated all the wounded and could refold intact [31, 38]. The partially damaged “Cassa di Risparmio” retreated with the third army. The Army Surgical Ambulances were evacuated with few losses [1, 38, 39]. Mobility had proved useful also in bad situations.

## Mount Grappa and The Piave. Malaria and Spanish Fever pandemic

With the front shortened on the Mount Grappa and behind the Piave river, medical services were reorganised and mobile surgical units were installed in sumptuous Venetian Villas along the west side of the Piave. They formed a network of highly specialized facilities, strategically located on the Venetian plain’s web of fine roads. During the June 1918 Austrian offensive, the surgical hospitals had to face an enormous volume of activity, but the morale of the entire Army had changed: enthusiasm and confidence prevailed and the decisive Solstice Battle (as that fighting was christened by the Italian poet Gabriele D’Annunzio) was won, opening to the final victory.

The military role of the Allied troops in Italy (three British and two French divisions) during the battle of June, although of value, was relatively marginal, and the presence of the Americans was limited to a single regiment (the 332nd Ohio) sent to Italy after the battle [40–42]. However, the medical help was important and highly appreciated [21, 42].

The British Red Cross motor ambulances, which had been in action already on the Isonzo, had generously continued to help the Italian ones in transporting the wounded from the front line to the surgical units [21]. The American Red Cross provided precious medical supplies and a fresh contingent of about one hundred American Red Cross Ambulances that had (along with the popular Rolling Canteens) a great moral impact [21, 42].

Following the Solstice Battle, surgical units were relieved from the pressure of war wounds but first malaria then the Great Flu epidemic in autumn, challenged both sides of the front. Soldiers with Spanish fever occupied hospital beds intended for the wounded. Mortality unrelated to war had rocketed in towns. The Consiglio Superiore di Sanità (Superior Council of Health) had initially minimized the risk, stating “there is no reason to worry particularly, as it is a Flu-outbreak, like in the year 1889–90” [43]. Later, measures of containment were undertaken, while hands hygiene, physical distancing and masks were advocated. Theatres, Cinemas and mass gatherings were closed. Religious functions were limited and funerals processions were forbidden. The Bishops encouraged the priests to inform the population during homilies about infection control, and to sanitize the benches after the service [44]. All this sounds incredibly familiar today.

## The final battle: victory

In November 1918 the mobile surgical units followed the Army’s victorious rush through the Friuli plains and up the Trentino valleys. Mobility had become important once again. During the pursuit and following the armistice they also cared for the destitute populations, ravaged by one year of Austro-German occupation, thus anticipating the “humanitarian missions” that medical mobile units now often ensure. Between December 1918 and early 1919, the three Red Cross mobile surgical hospitals and few army surgical ambulances returned home, and medical professionals regained civil practice to continue the challenge of the flu pandemic.

## Results and final appraisal

Following the war, the de-militarized surgeons reappraised the huge amount of clinical data that had been systematically collected throughout the war. Baldo Rossi and collaborators published a book on the Città di Milano’s 5497 operations, which became a war surgery reference textbook [18]. *Il giornale di medicina militare* reported a detailed experience of 1478 abdominal operations of the 4th Army Ambulance [33]. *Rivista ospedaliera* did the same for the 3rd Ambulance and some volumes reviewed the clinical series of the 6th Ambulance [45–48]. The official report by the Italian Red Cross at the end of the war lists more than 7.000 operations done in the three Red Cross surgical mobile hospitals only [49].

These, along with other sparse reports from the other units, lead to estimate not less than 20,000 wounded treated in the mobile units. The survival rate among the operated abdominal wounds ranged between 38 and 40%. An incredible achievement, especially considering the pre-antibiotic era, and on patients otherwise condemned to die.

According to ICRC War Surgery Textbook (2013), “post-operative mortality (for abdominal wounds) has decreased from around 67% in the late stages of World War I to 25% in World War II, down to 12% for US medical services in Korea and 8.5% in Viet Nam. Various contemporary studies report between 10 and 15% mortality. Only well-structured military services with forward surgical teams and rapid evacuation of patients achieve lower rates”. A yellow box entitled “Old lessons for new surgeons”, now endorses the philosophy of the old interventionists and states: “when in doubt, look and see rather than wait and see” [50].

The indirect impact that WWI mobile units had on other surgical units, acting as road-openers and stimulating interventionism and specialization, it is more difficult to estimate. But the surgical knowledge accumulated during the war rebounded in civil practice and promoted techniques and procedures never attempted before. Many of the mobile unit protagonists shaped the Italian surgical panorama for decades.

In spite of the gigantic medical achievements of the wars that followed, the strategy of approach to mass war casualties still pivots around “treat on the spot” or “stabilize and evacuate” models, depending on the number of casualties, the war theatre and the specific competencies of surgical teams. Role 3 mobile surgical units, be their name Ospedali Chirurgici Mobili, M.A.S.H, Combat Support Hospitals, Field Hospitals or other, whether transported by ground or by air, always search adequate medical answers in ever changing scenarios by modulating complexity, standardization and flexibility.

Present mobile surgical units, conceptually not so different from their WWI ancestors, are precious in war operations, peace keeping missions, humanitarian emergencies and natural disasters. Big helicopters deposit high-tech surgical containers in remote settings and guarantee prompt evacuation of stabilized patients; movable medical units care for refugees or support stable hospitals overwhelmed by pandemics; gigantic surgical lorries can be sent to disaster scenes or in underserved rural areas for itinerant surgery. They are, after all, only modern variations of the small and romantic, slow but reliable Fiat 15-ters, that climbed and descended the endless Alpine hairpin roads to deposit their operating tents just behind the frontline.
